# Chemical Mixtures: Considering the Evolution of Toxicology and Chemical Assessment

**DOI:** 10.1289/ehp.6987

**Published:** 2004-10-21

**Authors:** Emily Monosson

**Affiliations:** Community Science and Environment Program, Mount Holyoke College, South Hadley, Massachusetts, USA

**Keywords:** chemical mixtures, chemical regulation, mixtures assessment, risk assessment

## Abstract

The assessment of chemical mixtures is a complex topic for toxicologists, regulators, and the public. In this article the linkage between the science of toxicology and the needs of governmental regulatory agencies in the United States is explored through an overview of environmental regulations enacted over the past century and a brief history of modern toxicology. One of the goals of this overview is to encourage both regulators and scientists to consider the benefits and limitations of this science–regulatory relationship as they tackle existing issues such as chemical mixtures. It is clear that *a*) over the past 100 years chemical regulation and toxicologic research, have in large part, shared a common emphasis on characterization and regulation of individual chemicals. But chemical mixtures have been, and continue to be, evaluated at hazardous waste sites around the United States. For this reason the current U.S. Environmental Protection Agency guidelines for chemical mixtures assessment are also reviewed. These guidelines highlight the current practice of mixtures assessment, which relies primarily on the existing single-chemical database. It is also clear that *b*) the science and assessment of chemical mixtures are moving forward through the combined efforts of regulatory agencies and scientists from a broad range of disciplines, including toxicology. Because toxicology is at this exciting crossroads, particular attention should be paid to the forces (e.g., public demands, regulatory needs, funding, academic interests) that both promote and limit the growth of this expanding discipline.

Toxicology is the workhorse science of numerous industries and regulatory agencies. By providing a more complete understanding of the toxic effects of chemicals, toxicology has also provided many societal benefits. Toxicology is used in the characterization and development of standards for regulation of natural and produced chemicals, ranging from those commonly used in food production to chemicals that may contaminant soil, water, or sediments at hazardous waste sites. It has been noted that most 20th-century toxicologic studies were primarily concerned with the effects of individual chemical exposures, laying the groundwork for the development of toxicology as a “single-chemical science.” However, we are seldom if ever exposed to single chemicals; whether it is through our diet, pharmaceuticals, air, or our drinking water, we are exposed to mixtures of both anthropogenic and naturally occurring chemicals.

Members of communities located adjacent to hazardous waste sites or in industrial cities where they may receive exposure to hazardous chemicals through multiple sources are perhaps most acutely aware of their potential exposure to chemical mixtures. Often, in the United States, characterization of a hazardous waste site reveals multiple chemicals from multiple pathways resulting in a complicated matrix of chemicals and concentrations. For assessment purposes, the U.S. Environmental Protection Agency (EPA) defines chemical mixtures as either *a*) simple mixtures, containing “two or more identifiable components but few enough that the mixture toxicity can be adequately characterized by a combination of the components toxicities and the components interactions” or *b*) complex mixtures containing “so many components that any estimation of its toxicity based on its components’ toxicities contains too much uncertainty and error to be useful” (U.S. EPA 2000).

Current methodologies for human health risk assessment commonly treat mixtures as simple mixtures, deriving the combined toxicity of individual components primarily from single-chemical studies. Community members active in monitoring waste site assessment and remediation question the adequacy of human health assessments based on a components approach. They are concerned that all chemicals may not be accounted for, or that potential interactions may not be considered in health risk assessments. This present project originated from these community concerns. The original goal was to review current mixtures assessment methodology. However, it soon became clear that mixtures assessment is an evolving discipline within toxicology.

Because researchers and regulators working to improve the human health assessment of chemical mixtures will likely build upon past regulatory and toxicologic methodology and technology, it is relevant to consider the past century of toxicology and chemical regulation and consider past goals and limitations in both the science and regulation of chemicals. Therefore, this overview touches upon the modern history of toxicology (defined here as postindustrial/postchemical revolution, beginning around the mid-19th century) and chemical contaminant regulation. Of the regulations reviewed below, the assessment methodology for chemical mixtures set forth in support of the Superfund Comprehensive Environmental Response, Compensation, and Liability Act of 1980 (CERCLA) is reviewed in some detail (CERCLA 1980). A brief discussion of the current status of mixtures evaluation and concluding remarks follow.

## Toxicology

Toxicology is an ancient practice through which naturally derived poisons were likely used for hunting, warfare, medicinals, and even intentional poisonings (Borzelleca 2001). Although toxicology’s beginnings as a scientific discipline may be traced back several centuries to practitioners such as Paracelsus or Orfila (Borzelleca 2001), this overview focuses on a more modern era of toxicology.

The chemical/industrial revolutions of the mid-18th and 19th centuries resulted in large-scale releases of naturally occurring chemicals into the environment, in addition to the production and release of new substances unlike any that had existed before. This rapid growth of chemical use provided the backdrop for the emergence of the many branches of toxicology that exist today, including pharmacology, pesticide toxicology, occupational toxicology, and environmental toxicology. The toxicology we practice today was built upon the knowledge and methodology developed by its early practitioners. For example, observations of occupational illnesses associated with chemical exposures were recorded early in the history of toxicology [e.g., as noted by Borzelleca (2001), diseases of miners were described by Ramazzini in 1705], and modern occupational toxicology advanced our understanding of occupational disease in the early-20th century through the work of Alice Hamilton, Ethel Browning, and others (Borzelleca 2001).

According to an essay by John Doull (2001), formal recognition of toxicology as an academic discipline occurred around 1959 with the advent of the first journal devoted to toxicologic and pharmacologic study, the first modern toxicology textbook, and the formation of the first Society of Toxicology (SOT) in 1961. Although these dates mark the formal beginnings of the academic discipline, toxicologic studies had been conducted throughout the early 20th century to characterize various chemicals, including chemical warfare agents, pesticides, and food products, with practitioners trained in other fields, including medicine, chemistry, and pharmacology (Borzelleca 2001; Doull 2001; National Research Council 1997). It was through the efforts of these scientists that toxicology developed into a rigorous scientific and academic discipline.

At the time of toxicology’s emergence as a discipline, according to Scala (1999), the main emphasis of the field was devoted primarily to chemical characterization and safety evaluation, rather than mechanistic toxicology. Scala’s observation is interesting considering that according to Borzelleca (2001), toxicology’s mechanistic basis developed early in the mid-19th century, along with concomitant advances in physiology. Perhaps the emphasis on characterization and safety evaluation of the early 20th century resulted from the influence of governments needs. Indeed, one “working hypothesis about the development of toxicology is that the discipline expands in response to legislation, which itself is a response to real or perceived tragedy” (Gallo 1996). As reviewed below, much of the early legislation in the United States (e.g., early 20th century) pertained to the control and effectiveness of individual chemicals used in food, drug, and pesticide formulation.

Toxicologists have long understood that chemicals interact in the body and that the kinetics and dynamics of chemicals, in addition to consideration of the biochemical status (e.g., nutritional, hormonal, and stress status), are fundamental to the science. For example, through study of the cytochrome-P450 system (CYP), we know that endogenous chemicals may affect the kinetics and dynamics of xenobiotics and vice versa (Parkinson 1996). Knowledge that chemical interactions may underlie many toxicologic outcomes is integral to the teaching and practice of toxicology. Pharmacologists, in particular, routinely consider the importance of drug interactions, and literature on drug interactions abounds (e.g., Burns and Conney 1974; Indiana University School of Medicine 2004).

And so, although a great majority of early toxicologic studies were devoted to the characterization of individual chemicals, it was not for ignorance of the importance of multiple chemical interactions. An early discussion of chemical interactions by Bliss (1939) defines three main categories of joint chemical action that are still relevant today: *a*) independent joint action, which refers to chemicals that act independently and have different modes of action (i.e., different mechanisms), such that the presence of one chemical will not impact the toxicity of another, and the combined toxicity can be predicted from knowledge of the independent chemicals; *b*) similar joint action, which refers to chemicals that cause similar effects often through similar mechanisms, and in this case how the presence of one chemical may affect the impact of another chemical (e.g., if two chemicals, A and B, act by combining with the same receptor in the body, the impact of B will depend on how much chemical A is present—its effect might be lessened or heightened if A is present), so, as with independent joint action, toxicity can be predicted with knowledge of the independent chemicals; and *c*) synergistic action, where

the effectiveness of the mixture cannot be assessed from that of the individual ingredients but depends upon a knowledge of their combined toxicity when used in different proportions. One component synergizes or antagonizes the other. (Bliss 1939)

The U.S. EPA defines synergism as “when the effect of the combination is greater than that suggested by the component toxic effects” (U.S. EPA 2000).

More than six decades after Bliss’s publication, however, few studies have addressed the interactive effects of chemical mixtures. A limited review of 151 papers published in 1992 suggested that some “95% of the resources in toxicology is devoted to single-chemical studies” (Yang 1994a). Of the toxicologic studies that do address chemical interactions, the most focus on either sequential exposure to two different chemicals (e.g., many CYP studies employ sequential exposures) or exposure to binary mixtures (Hertzberg and Teuschler 2002; Yang 1994a). Additionally studies designed to address mixtures of several different chemicals at environmentally relevant concentrations [i.e., either near lowest observed adverse effect level (LOAEL) or no observed adverse effect level (NOAEL)] have led to opposing conclusions. One study suggests that “as a rule” mixtures below the NOAEL should present no health concern (interactive effects were reported at the LOAELs) (Cassee et al. 1998), whereas another suggests that even low-level exposure to chemical mixtures may cause subtle biologic effects, some of which may not be detectable by current methods (Yang 1994b). Still another review found that although some studies “support the hypothesis that adverse effects are unlikely when the mixture’s components are present well below their individual thresholds,” it is “prudent to anticipate exceptions to the rule” (Seed et al. 1995). Over the past 15 years, several studies with chemical mixtures (e.g., hormonally active chemicals or pesticides) at concentrations near or below the no observed effect concentration (NOEC) or the NOAEL have reported potentially harmful biologic responses (Cavieres et al. 2002; Rajapakse et al. 2002; Welshons et al. 2003).

It is clear that interest and research on chemical mixtures have intensified over the past decade, as evidenced by review articles (Carpenter et al. 2002; Feron et al. 2002; Seed et al. 1995), meetings [e.g., the Agency for Toxic Substances and Disease Registry (ATSDR) International Conference on Chemical Mixtures, co-sponsored by several federal and international agencies], and programs sponsored by the U.S. EPA, the National Institute of Environmental Health Sciences (NIEHS), Centers for Disease Control and Prevention (CDC), and the ATSDR. Although neither the concept of chemical interaction nor the laws addressing mixtures are new [the U.S. EPA (1986) first issued guidelines for mixtures in 1986 in support of CERCLA], the current interest and attention to mixtures are new. The focus on chemical interactions, particularly at environmentally relevant concentrations, is an important step toward advancing our understanding of the human health and environmental impact of mixtures. This new energy devoted to chemical mixtures inaugurates an exciting era in the evolution of toxicology.

## Chemical Regulation in the United States

The chemical control laws reviewed below, which evolved during the past 100–150 years, range from controls for pesticide residues to allowable concentrations of chemicals in surface or drinking waters. And, most focus on control of individual chemicals. Although some processes by which the toxicity of whole effluents are evaluated as complex mixtures, the focus of this overview is the body of laws designed to protect human health, which relies primarily upon evaluation and control of individual chemicals.

The laws are discussed in chronologic order (for the most part) and primarily focus on control of the environmental release of chemicals, except for the Occupational Health and Safety Act (OSHA) of 1970 (OSHA 1970). OSHA was included because some of the first methods for evaluating risk from multiple chemicals developed in the field of occupational health.

### Federal Food, Drug and Cosmetic Act.

One of the first chemical control acts, the Drug Importation Act of 1848, although short-lived, was enacted to protect the public from importation of ineffective adulterated drugs (Worobec 1986). The development and sale of unregulated remedies and food preservatives prompted further regulation and provided motivation for studies on their health effects. Preservatives were tested by Harvey Wiley’s now infamous “poison squad”: U.S. Department of Agriculture (USDA) volunteers who ingested several commonly used food preservatives (e.g., formaldehyde) (Hutt and Hutt 1984; Wiley 1907). These studies resulted in the creation of early food and drug control laws, such as the Food and Drug Act of 1906. Our current Federal Food Drug and Cosmetic Act of 1938 (as amended; FFDCA) authorizes assessments of the safety of new drugs, food additives, and colors and specifies tolerance levels for pesticides and other chemicals that may occur in foods (FFDCA 1938). It was the 1958 Food Additives Amendment (FFDCA 1958) that set forth the Delany Clause, prohibiting the approval of food additives found to cause cancer in humans or animals. With the advent of the Food Quality Protection Act of 1996 (FQPA; discussed below), the FFDCA was amended to eliminate the applicability of the Delany Clause to pesticides (FDA 2002; FQPA 1996).

From the earliest tests conducted by Wiley to many of the current toxicity tests, chemicals are tested and regulated on a single-chemical basis. Recent concerns for multiple or mixed pesticide residues in foods, however, have resulted in amendments to the FFDCA that address potential exposure pesticide mixtures in foods (FQPA 1996), as discussed below.

### Federal Insecticide, Fungicide and Rodenticide Act.

Before the 1970s, the USDA’s Federal Insecticide, Fungicide and Rodenticide Act (FIFRA) of 1947 was essentially geared toward protecting consumers from ineffective products (FIFRA 1972; Worobec 1986). The act was later amended several times, with the 1972 amendments providing the basis for current pesticide regulation. Jurisdiction of the act transferred from the USDA to the U.S. EPA in 1970. The changes in FIFRA beginning in the 1970s, along with the creation of the U.S. EPA, likely boosted the expanding field of environmental toxicology, with environmental toxicologists adapting existing methodology and designing new methodologies to fulfill the regulatory requirements to characterize individual chemicals (e.g., Rand and Petrocelli 1985).

### Food Quality Protection Act.

Together, the FFDCA and FIFRA regulate a large share of chemicals to which humans are exposed through their foods, drugs, and agricultural practices, primarily by setting tolerances and allowable concentrations for individual chemicals. Recent amendments to both FIFRA and the FFDCA via the FQPA depart from this practice by setting health-based standards for aggregate exposures (e.g., all dietary and residential exposures) to similar-acting pesticides in foods (FQPA 1996; U.S. EPA 2003a). The FQPA is one of the first attempts by the U.S. government to develop pesticide tolerances based on their potential for combined toxicity. (The methodology is discussed briefly later in this overview.) Fully addressing this goal will likely require development of new techniques to address more complex combinations (e.g., combined exposure to organophosphates and arsenic would not be addressed using the current approach).

### Clean Air Act.

The Clean Air Act (CAA) of 1955 [CAA 1955; summarized in U.S. EPA (1993a)] was created to ameliorate increasing smog problems, as exemplified by the Donora Smog of 1948 (Davis 2002). Although the early legislation mainly provided funds for air quality research rather than setting limits for pollutant releases, subsequent amendments eventually provided the U.S. EPA with an opportunity to develop national standards protective of human health and welfare. The 1970 amendment, for example, authorized the U.S. EPA to set forth National Ambient Air Quality Standards (NAAQS). The U.S. EPA issued its first six standards that specified allowable releases of the chemicals in 1971 (Worobec 1986). Before development of a new NAAQS, the U.S. EPA must develop air quality criteria for each chemical, detailing the scientific rationale for regulation. Under the CAA the U.S. EPA may consider the cumulative impact of chemicals from multiple sources and is directed to include information on air pollutants that “may interact with such pollutant to produce an adverse effect on public health or welfare” (CAA 1955; U.S. EPA 1993a; Worobec 1986).

### Clean Water Act.

Amendments to the early Federal Water Pollution Control Act of 1948, inspired by increasing awareness of the degradation of the nation’s waterways such as the release of kepone into the James River in Virginia, created the Clean Water Act (CWA) of 1972 for protection of surface waters (CWA 1972; U.S. EPA 1978; Worobec 1986). Under the CWA the U.S. EPA is directed to develop water quality criteria outlining permissible pollutant loadings for a particular water use and states are directed to set water quality standards that include limitations on individual chemicals [for a summary see U.S. EPA (2003b) and Worobec (1986)].

Additionally, under the CWA, states, territories, or authorized tribes are mandated to develop the total maximum daily load (TMDL) for water bodies that are impaired or do not meet the state’s water quality standard. Although by definition a TMDL refers to “the sum of the allowable loads of a single pollutant from all contributing [sources]” (U.S. EPA 2003c) when developing TMDLs, the U.S. EPA recommends that water quality, water chemistry, and cumulative impacts of individual chemicals and chemical mixtures be addressed through methods that may include single-chemical, whole-effluent toxicity testing (WET) and bioassays (U.S. EPA 1991). WET addresses complex chemical mixtures (U.S. EPA 1995, 2002); however, because they are designed primarily for the protection of aquatic health rather than human health, they will not be discussed further in this overview. Although requirement for TMDL is not new to the CWA, implementation of the TMDL is relatively recent, brought about in part by the legal action of citizens groups (U.S. EPA 2003d). It is unclear how often multiple chemical impacts are actually addressed.

### Safe Drinking Water Act.

We rely upon both surface water (protected by the CWA) and groundwater for drinking. The Safe Drinking Water Act (SDWA) of 1974 (amended in 1986 and 1996) was intended to protect drinking water and fill gaps left by the surface-water–focused CWA (SDWA 1974; U.S. EPA 2003e). Like much of the CWA, the SDWA also regulates on an individual chemical basis.

Amendments to the SDWA in 1996, however, now require “new approaches for studying the adverse effects of contaminant mixtures in drinking water” (U.S. EPA 1996). Concerns about complex mixtures of disinfection by-products (DBPs) in drinking water, for example, have led to research on the reproductive and developmental toxicity of DBPs (Simmons et al. 2002).

### Toxic Substances Control Act.

The Toxic Substances Control Act (TSCA) of 1976 (TSCA 1976) enabled the U.S. EPA to track industrial chemicals and to more collectively consider all uses of a particular chemical or chemical mixture with a focus on human health and environmental impacts (TSCA 1976). It was TSCA’s enactment that led to the ban on polychlorinated biphenyls (PCBs) (U.S. EPA 1979; Worobec 1986). Note, however, that the language of TSCA seems to refer only to commercial or industrial mixtures rather than mixtures resulting from separate processes involving several different chemicals (TSCA 1976; U.S. EPA 2003f).

### Occupational Safety and Health Act.

Because some of the first risk assessment techniques addressing chemical mixtures were developed for worker protection (OSHA 1970), OSHA is included in this overview. OSHA was designed in part to protect workers from exposure to harmful chemicals through establishment of standards and guidelines for individual chemicals. Before its enactment in 1970, industry, industrial hygienists, and workers recognized the need for worker protection. In the early 1940s, before any federal regulation, the American Conference of Governmental Industrial Hygienists (ACGIH) began developing what eventually became known as threshold limit values for exposure to industrial chemicals (ACGIH 2004).

Because industrial workers were seldom exposed to one chemical at a time, in 1963 the ACGIH established a methodology to address exposure to chemical mixtures (ACGIH 1963). The method was fairly straightforward: Unless there was evidence to the contrary, mixtures of chemicals that act on the same organ were to be treated in an additive manner using what they called the “additive mixture” formula (ACGIH 1963). OSHA gave the Occupational Safety and Health Administration the authority to adopt existing occupational exposure limits as legally enforceable exposure limits. In 1971 the ACGIH’s additive mixture formula was thus adopted as part of the “Z-table” permissible exposure limits (OSHA 1970, 1971). Note that workers may be exposed to relatively high chemical concentrations compared with many environmental exposures, which may have contributed to the ACGIH’s early focus on mixtures.

Together, the chemical control laws reviewed above make up the bulk of laws that govern the production and use of chemicals in drugs, food, consumer products, and the workplace, and control the release of chemicals into the environment. In large part, these laws focus on individual chemicals, and it is likely that before and after their enactment, a great deal of energy and funding were directed toward further use and development of single-chemical testing.

The rationale for a single-chemical approach in situations where multiple chemicals exist is partly based on the premise that chemical interactions either do not occur or are not toxicologically important at very low concentrations (e.g., below the NOAEL). However, a recent review of toxicologic studies designed to identify hormetic responses (defined as low-dose stimulation and high-dose inhibition) revealed that low-dose responses (below NOAEL) were “frequently encountered and [are] broadly represented according to agent, model and end point” (Calabrese and Baldwin 2001). Depending on the end point (e.g., endocrine alterations, tumor cell proliferation, organ proliferation, and immune alterations), a hormetic response may be considered beneficial, neutral, or adverse (Calabrese 2004). Recently, combinations of several estrogenic contaminants at concentrations below the NOEC were reported to greatly enhance estradiol 17β activity (Rajapakse et al. 2002). The potential for biologic effects below NOAEL, whether stimulatory or other, highlights the need, as discussed above, for research on potential interactions at these low concentrations.

### Comprehensive Environmental Response, Compensation, and Liability Act and Resource Conservation and Recovery Act.

The Resource Conservation and Recovery Act of 1976 (RCRA) and CERCLA were enacted to further reduce the release of industrial chemicals from operating and inactive facilities, respectively (CERCLA 1980; RCRA 1976). It is within these laws that the issue of chemical mixtures is most explicitly addressed.

The primary goal of RCRA is to protect humans and the environment from contaminants by empowering the U.S. EPA to control chemicals from their production to their disposal (or reuse) in both active and future facilities (RCRA 1976; U.S. EPA 1989a, 2003g). Notably, the guidance document for RCRA facility investigation allows for consideration of the additive health risk from multiple chemicals when assessing the site (U.S. EPA 1989b).

CERCLA, or Superfund, enacted in 1980, was designed to deal with hazardous waste that was for the most part not regulated under RCRA or any other environmental law—those chemicals released into the environment by closed and abandoned hazardous waste sites (CERCLA 1980; U.S. EPA 1989b, 2003h). The U.S. EPA Risk Assessment Guidance for Superfund acknowledges that “simultaneous subthreshold exposure to several chemicals could result in an adverse health effect” such that estimates based on single chemicals might underestimate the overall risk (U.S. EPA 1989b).

To aid risk assessors in the evaluation of chemical mixtures, the U.S. EPA developed guidance documents for the assessment of human health impacts from chemical mixtures (U.S. EPA 1986, 2000). Additionally the ATSDR, which conducts public health assessments at National Priorities List sites under CERCLA (U.S. EPA 1989b), has also initiated a chemical mixtures program, which includes the development of a guidance document for chemical mixtures assessment and several interaction profiles for commonly occurring chemical mixtures discussed below in this overview (ATSDR 2004).

The U.S. EPA’s Supplementary Guidance for Conducting Health Risk Assessment of Chemical Mixtures (U.S. EPA 2000) is a compilation of several different approaches to mixtures. Both this document and its predecessor include methodology cited by other agencies, including the Air Force Center for Environmental Excellence, which defers to U.S. EPA methodology under its risk assessment methods (HQAFCEE 2003), and the ATSDR. Selected sections of the U.S. EPA document are reviewed below. As with any guidance document, its use is at the discretion of the risk assessor.

## The U.S. EPA’s Supplementary Guidance for Conducting Health Risk Assessment of Chemical Mixtures

### Complex Mixtures

For complex mixtures, particularly those that consist of commonly produced commercial or industrial mixtures, the preferred method is to use mixture-specific toxicity data. This method is most appropriate for complex mixtures such as diesel fuels for which a great deal of toxicity data exist on the mixtures as a whole (e.g., World Health Organization 1996). PCBs are another mixture that at times has been treated as a complex mixture, with toxicity tests on specific PCB mixtures (e.g., Aroclor 1254 or Aroclor 1248), and at times treated as a simple mixture now that a number of the individual components have now been well characterized (e.g., Eisler 1986; Eisler and Belisle 1996; Safe 1990; Safe et al. 1985).

However, once released into the environment, depending on the mixture, previous use, route of release into the environment, and time in environment, the components of a mixture such as diesel fuel or PCBs may be altered (i.e., “weathered”). As a result, the mixture for which toxicologic data exist may not be identical in composition to the mixture in the environment. In these cases a risk assessor may opt to use a mixture that is considered “sufficiently similar” (U.S. EPA 2000). Unfortunately, toxicity data on whole mixtures or similar mixtures are seldom available for chemical mixtures other than the original commercial or industrial mixture.

### Simple Mixtures

In the absence of adequate data on a particular mixture, risk assessors may apply a “components” approach, where the data for each individual chemical are combined, most often in an additive manner. According to the U.S. EPA, should any information reveal the potential for interaction (i.e., synergism, antagonism), then these data “should be incorporated into the component-based approach. When there is no adequate interactions information, dose- or response-additive models are recommended” (U.S. EPA 2000). In the absence of evidence of chemical interaction, assumption of no interaction is the default approach, although, as discussed above, few data are available on chemical interactions, particularly at very low concentrations.

#### Dose addition.

Dose addition is suggested for chemicals that have similar toxicologic end points and toxicokinetics and for which either a lack of interaction is assumed or demonstrated through experimental data. In this case, similar toxicologic end points means chemicals that act by the same mechanism (e.g., chemicals bind with the same biologic receptor) or, if interpreted more broadly, damage the same target organ (Hertzberg and Teuschler 2002).

The dose addition methodology assumes that the potency of each chemical in the mixture can be calculated relative to each other or to one common chemical. Three methods of dose addition are the relative potency factor (RPF), the toxic equivalency factor (TEF), and the hazard index (HI). When mechanisms of action are well understood, RPF and TEF methods are recommended, whereas the HI is recommended in the absence of mechanistic data (U.S. EPA 1989b).

The RPF for a chemical is scaled according to its potency relative to an index chemical, a select chemical that is well characterized toxicologically and considered representative of the chemicals in the mixture. Chemicals that act through cholinesterase inhibition are suitable candidates for RPF methodology, and the U.S. EPA provides an example of RPF calculation using chlorophos as the index chemical. RPFs for all like-acting chemicals are scaled relative to chlorophos by calculating the ratio of a common measure of potency, such as the dose concentration (U.S. EPA 2000). The contribution of each individual chemical (in terms of chlorophos equivalent exposure) is determined by multiplying the RPF by either a calculated or measured quantity of each chemical [e.g., fruit and ingestion rates might be used to calculate exposure (U.S. EPA 2000)]. The predicted response is then quantitated using the sum of equivalent exposures and the dose–response curve for the index chemical. In this way, the RPF method provides a means for treating this kind of chemical mixture as a single chemical. In accordance with the FQPA, RPFs were recently calculated for a group of organophosphate pesticides (U.S. EPA 2001).

The TEF is considered by the U.S EPA to be a “special application” of the RPF. TEFs have been developed for several organochlorines that act primarily via the aryl hydrocarbon receptor, using 2,3,7,8-tetrachlorodibenzo-*p*-dioxin (TCDD) as the reference chemical (Van den Berg et al. 1998). The cumulative impact of the mixture is derived using the concentration of each chemical and the appropriate TEF (U.S. EPA 2000). There seems to be a fine line between those chemicals suitable for RPF and those suitable for TEF. According to the U.S. EPA, “the RPF method uses empirically derived scaling factors that are based on toxicity studies of the effect and exposure conditions of interest in the assessment,” whereas the TEF is based on “extensive mechanistic information that shows all the toxic effects of concern share a common mode of action” (U.S. EPA 2000).

With the HI, as with the RPF, there is an assumption that the impact of the chemicals is cumulative yet not interactive and involves adding up component concentrations. In this case, chemicals for which reference doses (RfDs) or reference concentrations (for inhalation) are available and that have been identified as chemicals of concern at the site are scaled using a common end point such as the RfD. The RfD is based on the NOAEL (derived through toxicity testing, including subchronic and chronic whole-animal studies) for the most sensitive end point, or the critical effect (U.S. EPA 1993b). This use of the critical effect assumes protection against any other toxic effects that may occur from exposure to the chemical. The contribution of each chemical component to the HI would then be calculated by dividing its concentration (*C*) by the RfD (*C*/RfD). This ratio produces a relative potency. These scaled concentrations are then added together to develop the HI for the whole mixture.

If the HI is equal to one, then the total exposure is interpreted as being equal to the mixture RfD (U.S. EPA 2000). Anything greater than one would represent a total concentration that is above the RfD, the interpretation of which would likely depend upon the situation and involve some judgment on the part of the risk assessor. An HI > 1 may prompt a risk assessor toward derivation of tissue- and mechanism-specific HIs to more accurately evaluate the mixture (U.S. EPA 1989b), although assessors are not required to do so. When using this approach, the U.S. EPA suggests that “exposure data should be at relatively low levels (near NOAEL), at which interaction effects are not expected” (U.S. EPA 2000).

#### Response addition.

Like dose addition, response addition is a “no-interaction” approach. The U.S. EPA suggests using response addition for chemicals that act so differently—the presence of one chemical in no way affects the toxicity of another chemical [U.S. EPA 2000; i.e., similar to independent joint action (Bliss 1939)]. The primary application of response addition has been limited to the assessment of chemical carcinogens, although the U.S. EPA guidelines suggest that this method could be applied to end points such as reproductive toxicity (which may all be different from each other but categorized as “reproductive end points”) (U.S. EPA 2000).

Response addition is used because each chemical in the mixture has a different critical effect rather than a common critical end point. The U.S. EPA suggests that dose–response curves developed through toxicity testing, with response “measured by the percentage ofexposed animals that show toxicity or the proportion of the population responding” (U.S. EPA 2000), could be used to estimate risk for each chemical and then summed to determine a cumulative risk. Because numerically adding risks will work only when there are low individual risks, this method would apply only to chemicals present in small concentrations.

When applying additive methodology, if all chemicals in a mixture do not contribute to toxicity, or if an antagonistic interaction occurs, an assumption of additivity could produce an overestimate of risk. Similarly, synergistic interactions might result in an underestimate. According to the U.S. EPA, “additivity assumptions are expected to yield neutral risk estimates” (U.S. EPA 1986).

The U.S. EPA’s MIXTOX database was intended to serve risk assessors and others as a database for interactive effects of chemicals. However, when first compiled, the database consisted primarily of binary studies and included only a small fraction of the potential chemical combinations that could exist at hazardous waste sites (Hertzberg and MacDonell 2002). An analysis of the database revealed that approximately 25% of the studies demonstrated “consistent synergism,” whereas the majority of studies showed mixed interactions, where a chemical pair might show synergy in one study and some other interaction in another (Hertzberg and MacDonell 2002). The authors of that study suggested that these differences might result from differences in the timing or sequence of chemical exposure, observations of different target organs, or different end points. Addressing only these issues (e.g., timing, concentration, and end points) with several different chemicals presents a potentially overwhelming task for toxicologists using traditional methodology.

#### Interaction-based Hazard Index method.

The interaction-based HI method is based on the HI but is designed to incorporate interaction data from binary testing to modify the HI. According to the U.S. EPA (2000), the method was developed explicitly to use binary interaction data and assumes that binary interactions reflect most of the possible interactions of the mixture. For example, a chemical combination of two chemicals A and B might affect the metabolic function of the liver and the heart, respectively. Chemical B is detoxified by the liver. If the liver is affected by chemical A, and its ability to detoxify chemical B is reduced, then combined exposure to these chemicals would result in enhanced toxicity, or a synergistic interaction. The influence of a third chemical C would then be assessed according to its binary interactions, first with A and then with B. The three-way interaction of A, B, and C would not be addressed explicitly but would be assumed to be not as significant as the two-way interactions.

The interaction-based HI method is modified by a “weight of evidence” evaluated by the risk assessor. The weight of evidence is dependent upon data available within the binary mixtures database and requires some judgment by the risk assessor in determining the nature of the chemical interaction, such as direction of the interaction (e.g., synergistic, antagonistic), plausibility that the interaction will occur, and the potential relevance of the interaction to human health (U.S. EPA 2000). If available, the magnitude of interaction would be incorporated as well.

Recently, the U.S. EPA conducted an evaluation of the application of dose addition for noncarcinogens and response addition for carcinogens, using the Comprehensive Environmental Response, Compensation, and Liability Information System database (U.S. EPA 2004a). From a limited review of 10 Records of Decision (full assessments were not always available), they concluded that “U.S. EPA practice is consistent with its own guidance regarding chemical mixtures assessment” (U.S. EPA 2004b). Mixtures assessments by the U.S. EPA and other agencies merit expanded analysis.

This brief overview of chemical regulation, toxicology, and the U.S. EPA guidance document brings up several important considerations in mixtures assessment: *a*) few data exist on many chemical mixtures; *b*) of those that do exist, most evaluate binary combinations; and *c*) few studies address chronic exposure to low concentrations of chemicals. As a result, the U.S. EPA guidance is to some extent constrained by these data limitations. As noted by members of the SOT–Society of Environmental Toxicology and Chemistry (SOT-SETAC) Working Group,

current methods of conducting chemical mixtures health risk assessments were developed to use available experimental data. . . These methods generally rely on default assumptions whose validity is unknown. (Teuschler et al. 2002)

## Current and Future Research on Chemical Mixtures

Although the concept of chemical interaction is not new, a new focus on mixtures has arisen in all areas of toxicology and regulatory policy in the United States. The need for improved chemical and toxicologic data and methodology for chemical mixtures to which the public is exposed has resulted in several initiatives by U.S. governmental agencies and researchers over the past decade. For example, the ATSDR developed a guidance for chemical mixtures that is fairly similar to the U.S. EPA guidance, although the ATSDR seems to place greater emphasis on physiologically based pharmacokinetic (PBPK) and pharmacodynamic (PBPD) modeling. The ATSDR has also developed, through literature review, interaction profiles for nine commonly occurring mixtures (e.g., one profile is for arsenic, cadmium, chromium, and lead) (ATSDR 2002; Hanson et al. 1998). The profiles, although often reliant on binary combinations, provide a detailed bibliography and literature review on selected mixtures and may at least highlight mixtures that depart from the assumption of additivity.

Additionally, other agencies such as the NIEHS, National Toxicology Program, and National Institute for Occupational Safety and Health have also begun programs to characterize exposures, develop biomarkers, and evaluate environmentally relevant mixtures (Bucher and Lucier 1998). A combined effort by the SOT-SETAC working group set forth three key ideas for future efforts: *a*) studies of mixtures should use low doses (e.g., below NOAEL); *b*) researchers should employ collaborative efforts; and *c*) researchers should explore novel approaches and technologies, noting that traditional animal-based toxicologic techniques are inadequate for such a complex issue (Teuschler et al. 2002).

In light of recent studies demonstrating endocrine disruption at concentrations below NOAELs or NOECs (Cavieres et al. 2002; Payne et al. 2000; Welshons et al. 2003), potential for hormetic stimulation (Calabrese 2004; Calabrese and Baldwin 2001), and cumulative impacts (Rajapakse et al. 2002), improving our understanding of chemical interactions at levels below NOAEL is clearly an important goal.

Collaborative efforts should involve not just toxicologists and their traditional collaborators (e.g., pharmacologists, epidemiologists, ecologists) but also other disciplines that work with complex systems, such as mathematics and physics. Additionally, new research approaches could incorporate community knowledge; for example, health surveys conducted by community members in collaboration with public health professionals might be used in addition to oral histories collected by agencies such as the ATSDR or the Occupational Safety and Health Administration. Including a community perspective (e.g., cultural practices, endemic diseases) may help to broaden our awareness of both chemical exposure and the public health impacts of chemical mixtures.

Toxicologists and others such as those working in genetics, molecular toxicology, or the newly developing field of toxicogenomics (Feron et al. 2002; Thomas et al. 2002) may soon be able characterize a chemical’s effect at the genetic level. However, chemicals may affect several different physiologic systems, and many of the body’s systems interact with each other (e.g., neuroendocrine, immune, and reproductive) (Carpenter et al. 1998) such that the biologic relevance or health impacts of these changes may sometimes be difficult to interpret. Consideration of chemical mixtures adds yet another layer of complexity.

Although one approach to reduce complexity may be to prioritize effects thought to be reliable predictors of health (e.g., selection of a critical end point), any prioritization must be flexible because that knowledge is limited by the current body of research. For example, although laboratory researchers had reported a range of endocrine or neuroendocrine toxicity in fish and wildlife for decades (Colborn and Clement 1992), many of these end points were not considered for human toxicity testing of industrial chemicals or standards development until the 1990s. After amendments to the SDWA and the passage of the FQPA, the U.S. EPA was directed to develop a screening program to identify the effects of chemicals on the endocrine system (U.S. EPA 1999). The Endocrine Disruptor Screening and Testing Advisory Committee was initiated to provide recommendations for the development of a screening program that would serve to provide information on endocrine-disrupting chemicals for regulatory application (Ankley et al. 1998; U.S. EPA 1999).

An improved database on individual chemical toxicity developed using new techniques or end points (e.g., genetics, endocrine testing) may improve the assessment of individual chemicals and aid in the development of computer models. Models designed to predict the effects of single chemicals (or drugs), including PBPK and PBPD modeling, have been under development for years. These models may eventually be useful for mixtures assessment (Bond and Medinsky 1995; Bucher and Lucier 1998), or at the very least, for predicting dose-dependent interactive effects. Currently, these models rely upon data derived from species-specific studies with single chemicals or, at best, binary combinations. However, a recent analysis using the PBPK framework with the common mixture of benzene, toluene, ethylbenzene, and xylene (e.g., BTEX) concluded that using mechanistic data derived from experiments with binary interactions could be extrapolated to more complex mixtures and different mixtures (Haddad et al. 2000). PBPK models may also be applied to the evaluation of metabolic interactions of certain chemical mixtures and elucidation of interaction thresholds (Dobrev et al. 2002).

Although these models offer some promise toward understanding and possibly screening for interactive effects of chemical mixtures (perhaps serving as a guide for more detailed studies), a great deal of validation will be necessary before they can be applied to chemical mixtures assessment. Presently, it is questionable if a computer model can fully represent the complexity of interactions of multiple chemicals in biologic systems.

At the other end of the methodologic spectrum are health-based approaches that implicitly consider the combined impact of chemicals on human health. These approaches include geographic/epidemiologic techniques, often focusing on a particular location (or set of locations) rather than on any particular mixture (Carpenter et al. 2001; Gilbertson et al. 2001; Nuckols et al. 1994). For example, Carpenter et al. (2001) studied the incidence of endocrine disease (e.g., thyroid and genital diseases) in areas of concern, areas where the potential for exposure to a suite of chemical contaminants exists, and found increased incidences of selected diseases. These studies are not designed to link cause and effect and currently are not directly applicable to current regulatory practices, but they may serve as a “hypothesis-generating exercise that will help sharpen the focus of research” (Elliot et al. 2001). Perhaps regulatory practices in the future may consider such studies. Another approach that integrates the effects of chemical mixtures is biomonitoring. For example, employees working at large waste disposal sites were found to have an increased incidence of chromosomal abnormalities compared with a control group (Fender and Wolf 1998).

Recently, the Pew Environmental Health Commission called for strengthening the nation’s public health system through several avenues, including merging the ATSDR with the National Center for Environmental Health in addition to providing federal support to improve community health monitoring to track diseases that may be caused by environmental contamination (Pew Environmental Health Commission 2001). A well-developed national repository of chronic diseases and developmental disabilities may aid toxicologists, epidemiologists, and others in the identification linkages between public health and contaminant exposure.

Finally, the Framework for Cumulative Risk Assessment set forth by the U.S. EPA in 2003 (U.S. EPA 2003i) moves even further away from single-chemical approach toward a multiple stressor approach (with stressor defined as “any physical, chemical or biological entity that can induce an adverse response”). Communities located near hazardous waste sites or heavily industrialized areas may be the ultimate integrators of the cumulative impacts of chemical mixtures. In some cases, these communities and the agencies responsible for evaluation and remediation of these sites have reached an impasse because of issues resulting from chemical mixtures. Progress toward addressing mixtures will depend upon creative and perhaps radically different approaches by regulators, toxicologists, and those with whom they collaborate. Under the cumulative risk initiative the U.S. EPA has worked with several communities to address the cumulative impacts of health hazards. For example, work with community groups from Cook County, Illinois, and Lake County, Indiana, was initiated after concerns were expressed about several different toxic emissions (e.g., dioxins, mercury, lead, and cadmium) from several proposed or existing incinerators (U.S. EPA 2003i).

## Conclusion

The goal of this overview was to survey both the science and regulatory history of chemical toxicants and to consider the relationship between the two. There is a shared 100- to 150-year history of single-chemical regulation and single-chemical research in the United States. Although consideration of chemical mixtures is not new to either realm, there is currently, and has been for the past decade, a greater emphasis on development of both scientific and regulatory methodology to improve our ability to evaluate the human and environmental health impacts of chemical mixtures (although only human health was addressed here).

When developing new methodology, new regulatory guidelines, or a new science, it may be useful to consider the forces acting to shape them (e.g., the existing knowledge base, available technology, funding, research opportunities, academic and government needs). Toxicology and chemical regulation are closely linked, and it is of interest to consider whether early chemical regulations encouraged the growth of a single-chemical science or if the existing toxicologic methodology and database moved regulators to consider controlling individual chemical exposures. If the single-chemical focus developed as a response to regulatory needs, what were the costs and benefits from that relationship? What were the limitations? More recently, for example, one might consider how the regulatory policy shift toward identification of endocrine disruption has influenced toxicologic research.

For various reasons, including regulatory requirements, improved technology and methodology, and perhaps public pressure, the challenge of chemical mixtures is shifting some part of toxicology away from the single-chemical model toward a different model that embraces multiple chemical exposures. Although there clearly remains a need for single-chemical toxicology in support of current public, regulatory, and industrial needs, there is also a need to develop mixtures methodologies. The knowledge derived from these two approaches can also inform each other, with single-chemical mechanistic and genetic data potentially useful in mixtures research, and an improved understanding of how combinations of multiple endogenous and foreign chemicals behave in the body contributing to better characterization of the toxic effects from single chemicals.

Toxicology and chemical regulation are at an exciting crossroads. Although this overview touched upon only past practices, its intent was to provoke both scientists and regulators to consider how their respective disciplines might influence one another and how they might influence the development of a model that integrates both single- and multiple-chemical approaches.
